# Application of Behavior Change Techniques in a Personalized Nutrition Electronic Health Intervention Study: Protocol for the Web-Based Food4Me Randomized Controlled Trial

**DOI:** 10.2196/resprot.8703

**Published:** 2018-04-09

**Authors:** Anna L Macready, Rosalind Fallaize, Laurie T Butler, Judi A Ellis, Sharron Kuznesof, Lynn J Frewer, Carlos Celis-Morales, Katherine M Livingstone, Vera Araújo-Soares, Arnout RH Fischer, Barbara J Stewart-Knox, John C Mathers, Julie A Lovegrove

**Affiliations:** ^1^ Hugh Sinclair Unit of Human Nutrition Department of Food and Nutritional Sciences University of Reading Reading United Kingdom; ^2^ Institute for Cardiovascular and Metabolic Research School of Biological Sciences University of Reading Reading United Kingdom; ^3^ Division of Applied Economics, Marketing and Development School of Agriculture, Policy and Development University of Reading Reading United Kingdom; ^4^ School of Life and Medical Sciences University of Hertfordshire Hatfield United Kingdom; ^5^ School of Psychology and Clinical Language Sciences University of Reading Reading United Kingdom; ^6^ Applied Social Sciences School of Natural and Environmental Sciences Newcastle University Newcastle Upon Tyne United Kingdom; ^7^ Human Nutrition Research Center Institute of Cellular Medicine Newcastle University Newcastle Upon Tyne United Kingdom; ^8^ Institute of Health and Society Newcastle University Newcastle Upon Tyne United Kingdom; ^9^ Marketing and Consumer Behaviour Group Wageningen University Wageningen Netherlands; ^10^ Division of Psychology Faculty of Social Studies University of Bradford Bradford United Kingdom

**Keywords:** behavior, behavior change technique, personalized nutrition, dietary management, nutrition, health, Web-based

## Abstract

**Background:**

To determine the efficacy of behavior change techniques applied in dietary and physical activity intervention studies, it is first necessary to record and describe techniques that have been used during such interventions. Published frameworks used in dietary and smoking cessation interventions undergo continuous development, and most are not adapted for Web-based delivery. The Food4Me study (N=1607) provided the opportunity to use existing frameworks to describe standardized Web-based techniques employed in a large-scale, internet-based intervention to change dietary behavior and physical activity.

**Objective:**

The aims of this study were (1) to describe techniques embedded in the Food4Me study design and explain the selection rationale and (2) to demonstrate the use of behavior change technique taxonomies, develop standard operating procedures for training, and identify strengths and limitations of the Food4Me framework that will inform its use in future studies.

**Methods:**

The 6-month randomized controlled trial took place simultaneously in seven European countries, with participants receiving one of four levels of personalized advice (generalized, intake-based, intake+phenotype–based, and intake+phenotype+gene–based). A three-phase approach was taken: (1) existing taxonomies were reviewed and techniques were identified a priori for possible inclusion in the Food4Me study, (2) a standard operating procedure was developed to maintain consistency in the use of methods and techniques across research centers, and (3) the Food4Me behavior change technique framework was reviewed and updated post intervention. An analysis of excluded techniques was also conducted.

**Results:**

Of 46 techniques identified a priori as being applicable to Food4Me, 17 were embedded in the intervention design; 11 were from a dietary taxonomy, and 6 from a smoking cessation taxonomy. In addition, the four-category smoking cessation framework structure was adopted for clarity of communication. Smoking cessation texts were adapted for dietary use where necessary. A posteriori, a further 9 techniques were included. Examination of excluded items highlighted the distinction between techniques considered appropriate for face-to-face versus internet-based delivery.

**Conclusions:**

The use of existing taxonomies facilitated the description and standardization of techniques used in Food4Me. We recommend that for complex studies of this nature, technique analysis should be conducted a priori to develop standardized procedures and training and reviewed a posteriori to audit the techniques actually adopted. The present framework description makes a valuable contribution to future systematic reviews and meta-analyses that explore technique efficacy and underlying psychological constructs. This was a novel application of the behavior change taxonomies and was the first internet-based personalized nutrition intervention to use such a framework remotely.

**Trial Registration:**

ClinicalTrials.gov NCT01530139; https://clinicaltrials.gov/ct2/show/NCT01530139 (Archived by WebCite at http://www.webcitation.org/6y8XYUft1)

## Introduction

### Emergence of Web-Based e-Resources

Improvement of health behavior relating to diet and lifestyle (eg, physical activity [PA]) is a key goal of studies aiming to reduce the incidence and progression of noncommunicable diseases (NCD). Chronic NCD such as cardiovascular disease (CVD), type II diabetes, and obesity carry heavy health care costs and are predicted to account for nearly three-quarters of global deaths in 2020 [1], with at least 2 million deaths each year currently associated with CVD in Europe alone [2]. Dietary and lifestyle factors play a key role in the progression and prognosis of many chronic NCD [3-5], and there is a continuing need to develop successful strategies to facilitate positive health-related behavior change. With the emergence of Web-based e-resources in electronic health initiatives, which offer cost-effective and fast delivery of health services [6], it is important to understand what drives behavior change in the context of these new digital environments.

### Evaluation of Web-Based Behavior Change Science

The science of health-related behavior change is complex and now requires reviewing owing to the large amount of research that has been conducted of late. Study designs are highly variable, and some interventions are more effective than others. New technologies such as mobile phones and other communication technologies are increasingly being used to deliver interventions, and this may influence behavior change technique (BCT) efficacy in ways we cannot yet predict. For instance, some meta-analyses have suggested that studies testing dietary and PA interventions that targeted fewer BCTs per individual were most effective [7].

In contrast, a meta-analysis conducted on computerized Web-based studies has suggested that the application of a greater number of BCTs to individuals was associated with greater effect sizes in successful interventions [8], although associations were not tested in the same individuals in Web-based versus face-to-face interventions, making comparison difficult. It may be the case that computerized studies offering less face-to-face support may benefit from the inclusion of greater numbers of BCTs that individuals can potentially pick and choose as appropriate. As Web-based studies offer access to greater numbers of individuals and are quicker and more cost-effective to deliver [6], it is likely that they will become increasingly popular with public health practitioners in the future. So it may also be necessary to formulate BCT strategies specifically for Web-based delivery methods.

In meta-analyses of dietary, PA, and smoking cessation (SC) interventions, the lack of or ambiguous recording of BCTs was highlighted, which hinders comparison and replication of different methodologies [7,8]. Until BCTs are properly recorded and BCT taxonomies are developed and used as a standard practice in studies seeking to change health behaviors, it will be difficult to assess BCT efficacy and to understand the psychological mechanisms underpinning intervention efficacy. BCT taxonomic frameworks are still being developed to enable a better understanding of dietary and other behavior changes to enable standardization of reporting, thereby providing a suitable basis for comparison, replication, and evaluation [9,10]. However, given the increase in Web-based delivery of health services, it is important to consider the development, use, and specification of BCTs in their design.

### Theory-Driven Application of a Web-Based Behavior Change Methodology

The taxonomy of BCT outlined in dietary and PA research by Susan Michie and her colleagues [9,10] was developed from earlier work by Abraham and Michie in 2008 [11]. An initial 26-item BCT taxonomy was derived from 72 intervention studies targeting diet and lifestyle behavior change. Michie et al developed well-validated BCT taxonomies for dietary behavior change, for example, the *Coventry, Aberdeen, and London-Refined,* or CALO-RE study [9], PA, and SC [10]. Michie et al’s BCT selection was derived from a number of theoretical standpoints [11] where BCTs were analyzed in terms of their deemed level of congruency or association with different important theoretical stances. These stances included control theory [12], which assumes that behavior is optimally changed by goal setting, self-monitoring, and evaluation; the Information-Motivation-Behavioral Skills model [13] and Theory of Planned Behavior [14], which focus on the provision of information on the link between behavior and health, health consequences of behavior, and others’ approval to bring about an intention to change; Social Cognitive Theory [15], where use of the social context is deemed necessary to understand barriers to change, provide support and encouragement for behavior change, and to learn from others; and Operant Theory [16], where reward-based learning occurs by identifying and using prompts and cues and by establishing routines to bring about good habit formation. Thus, the pan-European Food4Me study (N=1607) [17] provided an opportunity to use validated theory-driven BCT taxonomies to develop a BCT framework targeted at changing dietary and PA behaviors in an internet-based randomized controlled trial (RCT), with BCT selection for Food4Me being guided by this earlier theoretically driven work.

The overall aim of this paper was to articulate and describe the BCT Web-based methodology embedded in the structure and design of the Food4Me study and to explain why the BCT techniques were selected and for what purpose. Specifically, we aim to:

- Describe measurable BCTs embedded in the Food4Me study design from a validated BCT framework
- Demonstrate how the BCT framework was used in the development of standard operating procedures (SOPs) and training to maintain consistency across seven European countries
- Hypothesize as to the strengths and limitations of the BCT framework in the context of the Food4Me proof of principle (PoP) RCT
- Inform the use of this BCT framework in future studies of similar nature

Although psychological theories are described here briefly in terms of taxonomic development in general, it is beyond the scope of this methods paper to link BCTs to psychological theory.

## Methods

### Study Sample

The Food4Me PoP study was a 6-month, internet-based, 4-arm parallel, randomized controlled dietary intervention trial that took place in seven European countries (Germany, Greece, Ireland, the Netherlands, Poland, Spain, and the United Kingdom) from August 2012 to February 2014. Participants aged 18 to 80 years were recruited through their national recruitment center and undertook the study in the local language. Volunteers were excluded if they had no internet access, were suffering from chronic disease, were pregnant, lactating, or otherwise had special dietary requirements.

All participants signed Web-based consent forms at each of two screening stages, which were then returned electronically to the local study investigators for countersigning and archiving. Ethical approval for the Food4Me study (registered at Clinicaltrials.gov, NCT01530139) was granted by the local research ethics committee at each center.

### Study Design

**Figure 1:**
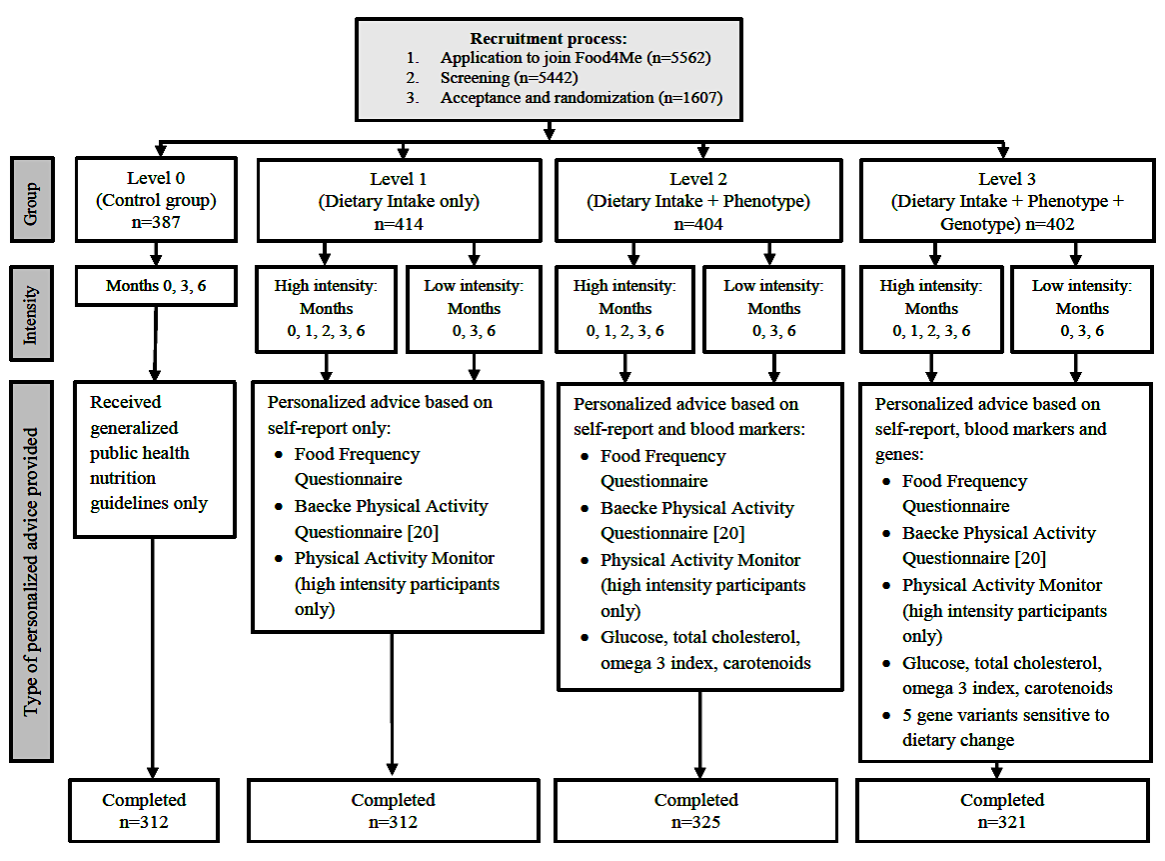
Process flow diagram for the Food4Me Proof of Principle study.

Participants were randomized to one of four arms (see Figure 1):

- Controls (level 0, L0) received currently accepted public health guidelines at months 0 and 3
- Levels 1, 2, and 3 (L1, L2, and L3, respectively) received personalized nutrition (PN) dietary advice at months 0 and 3 based on self-reported intake via Food Frequency Questionnaires (FFQs)

This PN advice took the form of three or four target nutrients to change and PA goals. L1 received dietary advice based on FFQ data alone, L2 on FFQ + phenotypic data from blood sampling, and L3 on FFQ + phenotypic + genotypic data. In addition, the frequency of advice was varied within each PN condition: low-intensity L1 to L3 participants received feedback at months 0 and 3 months, whereas high-intensity participants received additional feedback at months 1 and 2. Low-intensity L1 to L3 participants received basic PA advice and targets based on PA questionnaires collected at 0 and 3 months, whereas high-intensity participants also received feedback based on their PA monitor (TracmorD tri-axial accelerometer, Philips Consumer Lifestyle, The Netherlands [17]) data at months 0, 1, 2, and 3. All participants were required to use home kits to provide DNA samples at month 0 and blood samples at months 0, 3, and 6. Instructions for anthropometric measurements, DNA and blood sampling, and use of PA monitors were provided in hard copy form and were also available via video clips at the Food4me [18] website.

All participants completed a bespoke Dietary Change Questionnaire, designed to determine intention to change dietary behaviors [19], at their first measurement time point. The Baecke PA Questionnaire [20], a validated 16-item self-report tool to determine differences in three PA dimensions (habitual PA for work, sport PA during leisure time, and leisure time PA excluding sport), was administered at all data collection time points. The study design is described in full elsewhere [17].

All participants received a fully personalized report at the end of the study at month 6 in acknowledgment of their participation, which included their top three or four nutrient targets, PA goals, and blood and DNA results. This complex study design enabled comparisons over time between provision of general public health advice and personalized advice, between types of personalized advice delivered, and between high and low frequency (eg, intensity) of personalized advice provision [21].

### Behavior Change Technique Analysis

The analysis of BCTs used in the Food4Me study was carried out in three phases: (1) phase I (a priori): conduct a scoping review to identify theoretically appropriate BCT well-described in previous work [9,10] that could be applied to dietary and PA behaviors, in a remote or internet-based intervention context, for potential use in the Food4Me study; (2) phase II: develop a working BCT SOP for use by researchers in the PoP study, and train researchers at all Food4Me centers; and (3) phase III (a posteriori): review the BCT list on completion of the intervention study, and include any additional BCT utilized in the Food4Me study. Analyses for all three phases were carried out by Food4Me BCT researchers.

BCTs were reviewed for inclusion on the basis of their perceived capacity to support and promote change in dietary and other healthy behaviors utilizing the individual’s own motivations, capacity, and ability to change; the provision and delivery of dietary advice; and the quality of interactions supporting this provision.

BCTs were finally selected on the basis of how closely they aligned with the dietary and health goals of the study (for instance, in terms of the type, nature, and frequency of feedback to participants), on the basis of practicality (for instance, how far and how robustly they could be used remotely), and in terms of how easily they could be embedded in the provision of feedback, information, and advice.

## Results

### Behavior Change Technique Analysis Phase I— Food4Me Behavior Change Technique Identification

Phase I was carried out during the design phase of the Food4Me PoP dietary intervention study. Michie et al’s CALO-RE [9] and SC BCT [10] taxonomies were used to develop the Food4Me BCT framework. CALO-RE contains 40 uncategorized items, whereas the SC framework includes 43 items categorized into four functions, namely (1) motivation behaviors, (2) self-regulatory capacity or skill-related behaviors, (3) promotion of adjuvant (supporting) activities (eg, dietary advice), and (4) general aspects of interaction (eg, information gathering, delivery, and communication). Six BCTs from the SC BCT list that were not included in the 40-item CALO-RE BCT list were identified as being potentially useful for adaptation to the Food4Me dietary intervention, making 46 BCTs in total. The list of 46 BCTs was reviewed and agreed by six members of the Food4Me BCT research team based at Food4Me study centers at the universities of Reading, Ulster, Newcastle, and Wageningen.

### Behavior Change Technique Analysis Phase II— Food4Me Behavior Change Technique Development

On completion of the phase I analysis, the combined 46-item BCT framework was assessed to determine which BCTs were to be used in the Food4Me RCT (see Multimedia Appendix 1). At this stage, 11 items from the CALO-RE BCT list were judged appropriate when considering the constraints of a Web-based study in a remote setting.

Six items were adapted from the SC BCT list [10]: “emphasize choice,” “tailor interactions appropriately,” “assess current and past dietary behavior,” “assess current readiness and ability to change,” “assess past history of dietary change attempts,” and “assess adverse reactions.” These 6 items did not appear in the CALO-RE list; however, the research team considered that these BCTs were particularly appropriate for Web-based study designs and for studies conducted in remote settings. For instance, volunteers (1) received different types of advice depending on group allocation (tailor interactions appropriately); (2) were provided with choices of healthier foods (emphasize choice); (3) were assessed, and responses compared, at a number of time points (assess current and past dietary behavior), with readiness and ability measured by the Dietary Change Questionnaire (assess current readiness and ability to change); and (4) their adverse reactions were recorded throughout in line with clinical best practice (assess adverse reactions). Attempts were made to adapt the SC BCT texts for a dietary intervention where necessary and to align the adapted BCT text to reflect the commonality and underlying purpose of the BCT. For instance, “tailor interactions appropriately” [10] suited the Food4Me design where feedback was based on personal characteristics such as self-reported dietary intake, blood markers and genotype, and the text was included unchanged, whereas “assess current and past smoking behavior: assess amount smoked, age when started, pattern of smoking behavior” [10] was adapted to “Assess current and past dietary behavior: assess amount of food eaten and current and past patterns of food eaten,” as this was measured during the intervention. Multimedia Appendix 2 shows the changes made with rationales for the adapted text. The finalized revised list was then used to develop an SOP for use in all participating countries.

BCTs requiring more individualized or additional training resources and not in effect representing one single BCT but a set of them, such as BCT items 15 (prompting generalization of a target behavior), 36 (stress management or emotional control training), and 37 (motivational interviewing), were excluded. Items judged to require more in-depth or face-to-face interaction or resources beyond the scope of the study were excluded. Examples of excluded items are 23 (teach to use prompts or cues), 28 (facilitate social comparison), and 33 (prompt self-talk). BCTs with a negative inference, for instance, items 31 (prompting anticipated regret) and 32 (fear arousal) were excluded, as advice was designed to emphasize the benefits of following recommendations (eg, increasing intake of fruits and vegetables has been shown to reduce your risk of CVD) rather than focus on risk per se (eg, if you don’t eat enough fruits and vegetables you may be at greater risk of CVD). In this internet-based study, it was possible that the Web-based interface and associated lack of face-to-face support could have exacerbated any negative emotions on the part of the participant that the researchers would have been unable to monitor, control, or manage effectively. The rationale used for excluding BCT items is shown in Multimedia Appendix 3.

In summary, of the 46 BCT items previously identified, a total of 17 items were initially deemed appropriate to use when designing the Food4Me RCT and were included in the SOP during phase II. For practical reasons, it was decided to adopt the categorization framework developed for the SC program, as this was judged to be easier to communicate to all researchers and easier to use in practice in the SOP. The 17-item Food4Me BCT SOP was reviewed and agreed by the 6-strong Food4Me BCT research team.

### Behavior Change Technique Analysis Phase III— Food4Me Behavior Change Technique Poststudy Review

At the end of the study, the Food4Me SOP BCT was reviewed within the context of the intervention delivery. The 17 SOP BCT had initially been adopted across all centers, as these had been embedded in the design and implementation of the intervention study. A further 9 CALO-RE BCT had been adopted during the course of the study owing to the development of interim reports containing various types and levels of participant feedback for diet and PA. The interim report development had occurred in parallel with, or after, publication of the BCT SOP. This phase III analysis indicated that 26 BCTs were actually being used in the Food4Me dietary and lifestyle intervention, of which 20 came from the CALO-RE BCT list, and 6 were adapted from the SC BCT list (see Multimedia Appendix 1).

## Discussion

### Principal Findings and Comparison With Other Work

The identification of BCTs used in the Food4Me PoP study took place over three phases: identification of possible BCT for use in the Food4Me study (phase I: identification), development of an SOP (phase II: development), and review of the BCT used in the intervention (phase III: review). Initially, 46 BCTs were selected from validated BCT taxonomies [9,10] for possible inclusion, and 17 BCTs were selected for inclusion in the Food4Me PoP study SOP. At the end of the study, a further 9 BCTs were identified from the CALO-RE list in the final review as having actually been used by researchers after the development of the feedback reports. BCTs were largely embedded in the study design, which lent itself well to the development of a BCT SOP for use across all seven European study centers. This approach, for example, of a priori BCT review taking into account important contextual constraints on the delivery format and ad hoc a posteriori revision, is another form of approaching intervention development for adoption by future multicenter intervention studies where SOP may undergo further iterations and refinements in response to unanticipated needs emerging during the study.

In comparison with other dietary studies, the Food4Me PoP study had a higher number of BCTs embedded in its design. In Michie et al’s meta-analysis of interventions targeting improvements in smoking-related behaviors, dietary intake, and PA [7], dietary interventions included four to 19 BCTs, and the most successful interventions had fewer BCTs. This conclusion has been supported elsewhere [22]. However, the meta-analysis carried out by Thomas Webb et al on internet-delivered health interventions reported that more effective interventions were associated with greater numbers of BCTs [8]. There is still much work to be done to determine the exact nature of the relationship between the number of BCTs and efficacy of an intervention, which may be driven by any number of other factors, the assessment of which is beyond the scope of the current analysis.

Previous studies have usefully attempted to categorize the BCT taxonomy in terms of type of BCT category. For instance, the SC taxonomy [10] distinguished between motivation-based BCT, self-regulatory BCT, BCT providing adjuvant (supporting) activities, and BCT relating to general interactions (delivery, information gathering, and communication). This framework was crucial in helping us to describe the Food4MePOP study BCT framework and for identifying BCTs suitable for delivery of an internet-based intervention. For example, the SC BCT taxonomy included “before” and “after” comparisons, which have formed the basis of previous intervention studies, where feedback has been based on outcomes measured during the study. The SC taxonomy also included a BCT to monitor adverse reactions (eg, nicotine withdrawal). Reporting of adverse events (AEs) is considered the best clinical practice and is mandatory in clinical trials [23]. Dietary trials, including the Food4Me study, which include invasive measurements such as blood sampling in the home, should ideally aim to meet similar standards, even if recording of AEs is not compulsory. Finally, the categorization framework was particularly useful when communicating the BCT SOP to study researchers and for researcher training.

Three CALO-RE BCTs and five SC BCTs were subjected to varying degrees of adaptation for use in the Food4Me study; further scrutiny may be required to determine if altered BCTs are essentially the same as the original BCTs, or if the revised BCTs are distinct concepts in their own right. For instance, reporting of adverse *reactions* (eg, nicotine withdrawal) in the SC BCT may relate only to cause and effect as a direct result of the intervention target outcomes (eg, stopping smoking), whereas reporting of AEs appears broader and may relate to intervention outcomes (eg, excessive weight loss and reactions to recommended foods) and measurement factors (eg, blood-sampling in the home), both of which may hinder trial completion and prevent target outcomes from being achieved. Clinical best practice dictates that any study impacting on an individual’s health and well-being should include an overarching BCT for reporting AEs (including reactions), although this could be in addition to, or instead of, the adverse reactions to BCTs. BCTs were excluded if they were considered to be more appropriate for face-to-face interventions, which were beyond the scope of the study, or required additional resources that were incompatible with the original study design. In particular, BCTs that were thought to instigate negative emotions (eg, fear arousal and prompt anticipated regret) were avoided, in case they brought about adverse reactions that would be difficult to monitor or manage in an internet-based study.

Although some researchers have started to define BCT frameworks for use in intervention design (conceptual BCT-based design), many do not consider doing so in this way, with some key exceptions [24-26]. BCT analysis is still at an early stage with respect to dietary intervention studies and is not yet in a state where BCT descriptions may be linked to psychological constructs and its mechanisms [27,28]. The development of meta-analysis methodologies is ongoing and will not only contribute greatly to an understanding of the psychological mechanisms underpinning BCTs but also to an observable linkage with intervention efficacy [27]. However, such work is hampered by inadequate descriptions of study designs, failure to identify BCT a priori, or to monitor actual BCT use in interventions [9]. It is therefore recommended that future intervention design should incorporate a priori BCT identification, especially to aid the development of SOPs, and a posteriori BCT review, to ensure that all relevant BCTs have been captured and identified for future analysis in meta-analyses designed to determine such links, as the initially proposed BCT might change to better fit the context and individual needs. In this study, we are confident that the Food4Me study BCT framework has been well defined and categorized and will enable replication in the future.

To our knowledge, BCTs have not previously been described and categorized a priori for use in an internet-based PN intervention study of this nature, where participants were required to provide samples using home testing kits. Neither have they been used in the development of SOP for European multicenter research for a PN intervention study on this scale. As such, this a priori categorization combined with an a posteriori review of BCT in an internet-based, pan-European PN or PA RCT intervention is a novel use of the BCT framework taxonomy [9-11] and the first of its kind to do so.

### Strengths and Limitations

The process of defining the Food4Me BCT framework revealed a number of key strengths in our methodology. First, it enabled a clear understanding of the complex nature of the BCT framework used in an intervention where behavior change was the primary outcome. This is important: behavior change is poorly understood and difficult to predict in dietary and lifestyle interventions, so consistent and comparable use of methods that may contribute to our ability to determine drivers of behavior change is invaluable. A second important strength was that the development of the Food4Me BCT framework enabled us to use and test two established, evidence-based, theory-derived BCT taxonomies. CALO-RE and SC were found to be user-friendly and helpful in identifying target BCT and informing intervention design, development, and evaluation, although as Michie et al have acknowledged [9], there is still work to be done to develop these taxonomies further. Indeed, by combining the two taxonomies, the Food4Me study researchers were able to identify gaps in the CALO-RE taxonomy. These gaps were addressed by revisiting the SC taxonomy and by additional use of the SC categories. A third major strength of the Food4Me study approach was the creation and dissemination of a BCT SOP to be used by all recruiting centers, which helped to maintain consistency across seven European countries and provided the basis for researcher training. To our knowledge, this is a novel use of the BCT framework in a complex, pan-European, internet-based study. A final key strength was the incorporation of a three-phase process to define the Food4Me BCT framework, enabling a complete audit of BCTs at the study design, development, and completion stages. This mapping of the evolution of the decision-making process for the selection of BCTs, from conception and design through to execution, has the added advantage that it will contribute to the BCT meta-analysis process, as documentation does not always occur satisfactorily in this way, with either a priori or, in most cases, a posteriori recording and determination [7].

Some limitations were encountered. The BCT SOP was developed in parallel with other key aspects of the study (eg, interim reports) and was distributed before completion of the interim report piloting. This resulted in the choice of SOP BCT being dependent on design choices previously made in other elements of the study (for instance, blood collection processes and type or availability of other information on which advice was based), and this may have limited our ability to choose the most effective BCTs. Future researchers should attempt to design elements of the study likely to influence behavioral outcomes in advance of BCT analysis and before the start of the study. However, as we have demonstrated here, this is not always possible in practice, especially in complex multidisciplinary and multicenter experimental interventions with competing parameters. Second, in addition to the CALO-RE BCT, we used some SC-specific BCT to meet the needs of the Food4Me BCT framework; in some instances, we made alterations to existing BCT texts where the existing BCT did not fully apply to the specific needs of Food4Me. As a consequence of this, the meaning of the BCT may be slightly different from applications elsewhere, making comparison with other intervention studies difficult. Third, a number of elements of the Food4Me study, such as the interim report, incorporate several BCTs, which makes it difficult to assess the impact of a single BCT on study outcomes. Future research should investigate the effects of both single BCTs and BCT combinations, as combinations will typically be used in practice. The Food4Me results may provide insight into the latter.

### Future Recommendations

Our research has shown that BCTs can be usefully incorporated into the development of a complex dietary and PA Web-based RCT. It is recommended that literature-based lists, and possibly exploratory research, are used to provide clear justification for the inclusion or exclusion of BCTs in research designs. However, it should also be noted that a degree of pragmatism, which in this case was based upon study complexity, might be required in determining the number of BCTs to measure, especially where there is a lack of clear guidance within the literature about a recommended range of BCTs to measure. Finally, particularly in complex study designs, there should be sufficient flexibility to allow for additional BCT measures where necessary. Routine explicit description of BCTs used in research studies will help to enhance our understanding of BCTs for use in both specific and generalized situations and enable us to determine the optimal number and range of BCTs to incorporate into RCTs.

### Conclusions

Validated BCT taxonomies were helpful in developing the Food4Me BCT framework. Using an existing taxonomy to develop a BCT framework enables replication and comparison in future meta-analyses. The Food4Me framework will contribute to the future determination of psychological constructs and mechanisms underpinning behavior change and intervention efficacy. Categorization and description assisted the development of SOP and promoted consistency in experimental work. All BCT frameworks should be described and evaluated both a priori and a posteriori to aid replication and future analysis.

## Appendix

Multimedia Appendix 1 Coventry, Aberdeen, and London-Refined and smoking cessation behavior change techniques included in the Food4Me study.

Multimedia Appendix 2 Changes made to Coventry, Aberdeen, and London-Refined and smoking cessation Michie et al behavior change technique descriptions adapted for Food4Me on finalization of standard operating procedures.

Multimedia Appendix 3 Coventry, Aberdeen, and London-Refined behavior change techniques excluded from Food4Me, with rationale.

## Abbreviations

AE: adverse event
BCT: behavior change technique
CALO-RE: Coventry, Aberdeen, and London-Refined
CVD: cardiovascular disease
FFQ: Food Frequency Questionnaire
NCD: noncommunicable diseases
PA: physical activity
PN: personalized nutrition
PoP: proof of principle
RCT: randomized controlled trial
SC: smoking cessation
SOP: standard operating procedure
