# Supplementary material for: Application of Behavior Change Techniques in a Personalized Nutrition Electronic Health Intervention Study: Protocol for the Web-Based Food4Me Randomized Controlled Trial
Source: JMIR Res Protoc. 2018 Apr 9;7(4):e87. doi: 10.2196/resprot.8703 (PMC5913568; doi:10.2196/resprot.8703)
Supplement: Multimedia Appendix 1 [file resprot_v7i4e87_app1.pdf]

Multimedia Appendix 1. Coventry, Aberdeen, and London-Refined (CALO-RE) [9] and smoking cessation (SC) [10] behavior change techniques (BCTs) included in the Food4Me study.

BCT category	Food4Me code (CALO-RE[a] or SC[b] BCT[c] code [9,10])	BCT title [9,10]	Examples of where BCTs are used in Food4Me
Behavior and motivation	1 (1)	Provide information on consequences of behavior in general[d]	L0 (level 0; control group) public health nutrition guidelines
			L1-L3 baseline or interim reports: portion guidelines (eg, fruit and vegetables, wholegrain, dairy, oily fish, and red meat)
	2 (2)	Provide information on consequences of behavior to the individual[d]	L1-L3 baseline or interim reports: nutritionist’s message, physical activity (PA) messages
	3 (3)	Provide information about others’ approval[e]	L1-L3 baseline or interim reports: nutritionist’s message
	4 (13)	Provide rewards contingent on successful behavior[d]	L1-L3 baseline or interim reports: nutritionist’s message, PA messages
			L0-L3 PA monitor daily “green light” feedback (eg, green lights show in response to PA)
	5 (14)	Shaping[e]	L0-L3 PA monitor daily “green light” feedback (eg, increasingly challenging “green light” targets during the first 12 weeks)
	6 (19)	Provide feedback on performance[d]	L1-L3 baseline or interim reports: nutritionist’s message, dietary intake, blood markers (L2/L3 only) and genotype (L3 only), PA feedback, PA monitor results (high intensity only)
			L0-L3 PA monitor daily “green light”

			feedback (eg, green lights displayed in response to PA)
Behavior and self-regulatory capacity	7 (5)	Goal setting (behavior)[e]	L1-L3 baseline or interim reports: nutritionist’s message, dietary intake targets, PA targets
	8 (6)	Goal setting (outcome)[e]	L2-L3 baseline or interim reports: blood markers (eg, improve cholesterol levels by eating less saturated fat)
	9 (9)	Set graded tasks[d]	L1-L3 baseline or interim reports: anthropometrics (eg, try to lose 1-2 lb per week)
	10 (10)	Prompt review of behavioral goals[d]	L1-L3 interim reports: portion numbers, review of nutrient intakes, and PA
	11 (11)	Prompt review of outcome goals[d]	L1-L3 interim reports: anthropometrics, blood markers (eg, review of weight loss or cholesterol levels for L2 or L3 during study)
	12 (17)	Prompt self-monitoring of behavioral outcome[d]	L0-L3 PA monitor lights and data upload (eg, upload data every 2 weeks), anthropometrics (eg, take regular weight measurements)
	13 (7)	Action planning[e]	L1-L3 baseline or interim reports: setting nutrient-based or food targets (eg, eat more unsaturated fats or try to consume 1-2 portions of oily fish per week)
	14 (8)	Barrier identification or problem solving[d]	L1-L3 interim reports: nutritionist’s message (eg, researchers provided a list of barriers with suggestions on how to overcome these based on Dietary Change Questionnaire responses that individuals could adapt to personal circumstances)

	15 (26)	Prompt practice[e]	L1-L3 interim reports: targeted nutrient advice (eg, use less salt when cooking or at the table), targeted PA advice (eg, try to do 30 min of moderate to vigorous exercise 5 days a week)
Adjuvant activities	16 (29)	Plan social support or social change[e]	L1-L3 encouraged participants to contribute to the Food4Me web-based forum (high intensity participants only), where social support can be provided or sought
Interaction and delivery	17 (RD2)	Emphasize choice[f]	L1-L3 baseline or interim reports: nutritionist's message, nutrient- or food-based targets (eg, advice to use alternative sources of target nutrients to suit personal preferences)
	18 (RD1)	Tailor interactions appropriately[f]	L1-L3 baseline or interim reports: delivered feedback based on dietary intake, blood markers (L2 or L3 only), genes (L3 only), PA monitor feedback (high intensity participants only)
Interaction and information gathering	19 (RI1)	Assess current and past dietary behavior[f,g]	L1-L3 interim reports: delivered feedback on portion numbers and nutrient intakes for comparison with baseline report, highlighted differences in nutritionist's message
	20 (RI2)	Assess current readiness and ability to change[f,g]	L0-L3 screening questionnaire, health behaviors questionnaire, and Dietary Change Questionnaire
	21 (RI3)	Assess past history of dietary change attempts[f,g]	L0-L3 measured self-reported changes (eg, Food Frequency Questionnaire and Dietary Change Questionnaire) and objective changes (eg, blood markers)

	22 (RI4)	Assess adverse reactions[f,g]	L0-L3 recorded adverse events or reactions in accordance with clinical best practice
Interaction and communication	23 (21)	Provide instruction on how to perform the behavior[d]	L0-L3 provided tips on how to achieve the dietary and PA targets (eg, to increase your healthy fat intake, try having a handful of nuts as a snack)
	24 (40)	Stimulate anticipation of future rewards[d]	L1-L3 to encourage continued participation and data collection; baseline or interim reports with dietary and PA advice were promised at months 0 and 3; additional interim reports at months 1 and 2 were promised to high-intensity participants
			L0-L3 to encourage completion of the study; a final report was offered to all participants as a thank you for taking part, with personalized nutrition (PN) advice based on dietary intake, PA, blood markers and genotype (especially important for L0 who received no PN advice during the study)
	25 (27)	Use of follow-up prompts[e]	L1-L3 high intensity participants were offered PN reports at months 1, 2, 3, and 6
	26 (20)	Provide information on where and when to perform the behavior[e]	L0-L3 provided advice on when, for instance, dietary and PA changes could be implemented, for example, at snack times or in the evening

[a]CALO-RE: Aberdeen, and London-Refined.

[b]BCT: behavior change technique.

[c]SC: smoking cessation.

[d]BCT titles are sourced from Michie et al's CALO-RE taxonomy [9] at phase II.

[e]BCT added from Michie et al's CALO-RE taxonomy [9] at phase III.

[f]BCT titles are sourced from Michie et al's SC taxonomy [10] at phase II.

[g]BCT titles are adapted for Food4Me.

References

[9] Michie S, Ashford S, Sniehotta F, Dombrowski S, Bishop A, French D. A refined taxonomy of behaviour change techniques to help people change their physical activity and healthy eating behaviours: the CALO-RE taxonomy. Psychol Health 2011;26(11):1479-98. PMID: 21678185

[10] Michie S, Hyder N, Walia A, West R. Development of a taxonomy of behaviour change techniques used in individual behavioural support for smoking cessation. Addict Behav 2011;36(4):315-9.PMID: 21215528
