# Supplementary material for: Application of Behavior Change Techniques in a Personalized Nutrition Electronic Health Intervention Study: Protocol for the Web-Based Food4Me Randomized Controlled Trial
Source: JMIR Res Protoc. 2018 Apr 9;7(4):e87. doi: 10.2196/resprot.8703 (PMC5913568; doi:10.2196/resprot.8703)
Supplement: Multimedia Appendix 2 [file resprot_v7i4e87_app2.pdf]

Multimedia Appendix 2. Changes made to Coventry, Aberdeen, and London-Refined (CALO-RE) and smoking cessation (SC) Michie et al [9,10] behavior change technique (BCT) descriptions adapted for Food4Me on finalization of standard operating procedures (SOPs).

Food4Me BCT[a] code (CALO-RE[b] or SC[c] BCT code [9,10])	BCT [9,10]	Food4Me BCT (changes from source BCT [9,10] in italics)	Definition adapted for Food4Me (F4M; changes to source BCT [9,10] description in italics)	Comments
11 (11)	Prompt review of outcome goals[d]	Prompt review of outcome goals	Involves a review or analysis of the extent to which previously set outcome goals (eg, to *improve blood markers* or lose or maintain weight) were achieved. In most cases, this will follow previous goal setting (see technique 6[F4M8], goal setting-outcome) and an attempt to act on those goals, followed by a revision of goals and the means to attain them. Check that any instance does not also involve techniques 5[F4M7] (goal setting—outcome), 8[F4M14] (barrier identification or problem solving), 9[F4M9] (set graded tasks), or 10[F4M10] (prompt review of behavioral goals)	
12 (17)	Prompt self-monitoring of behavioral outcome[d]	Prompt self-monitoring of behavioral outcome	The person is asked to keep a record of specified measures expected to be influenced by the behavior change, *for example*, *blood glucose*, weight loss, and physical fitness. NB: It must be reported as part of the intervention, rather than only as an outcome measure. Check the distinction between this and techniques [sic] 16[F4M not used] (prompt self-monitoring of behavior)	BP was not measured in this study

17 (RD2)	Emphasize choice[d]	Emphasize choice	Emphasize volunteer choice within the bounds of evidence-based practice. *For instance, where recommendations include disliked foods, help volunteer to identify alternative sources of target nutrients to enable volunteer to follow dietary change guidelines*	Possible misinterpretation of original BCT
19 (RI1)	Assess current and past smoking behavior[e]	Assess current and past *dietary behavior*	No change	Possible misinterpretation of original BCT
20 (RI2)	Assess current readiness and ability to quit[e]	Assess current readiness and ability to *change*	Assess current level of motivation to *eat more healthily* and confidence in success	Possible misinterpretation of original BCT
21 (RI3)	Assess past history of quit attempts[e]	Assess past history of *dietary change* attempts	Assess *past and present dietary behavior change* attempts, including *barriers to adopting healthier behaviors measured at screening and at the end of the study*	Eating behaviors not measured before the study. Possible misinterpretation of original BCT
22 (RI4)	Assess withdrawal symptoms[e]	Assess adverse *events*	Assess the presence and severity of *adverse events that may impact on volunteer health during the intervention and on experimental outcomes*	Possible misinterpretation of original BCT
23 (21)	Provide instruction on how to perform the behavior[d]	Provide instruction on how to perform the behavior	Involves telling the person how to perform behavior or preparatory behaviors, either verbally or in written form. Examples of instructions include the following: how to *measure height and weight* (without *physically* showing the participant), instruction on suitable clothing, and tips on how to take action.	

			Showing a person how to perform a behavior without verbal instruction would be an instance of technique 21[F4M23] only. NB: Check whether there are also instances of techniques 5[F4M7], 7[F4M13], 8[F4M14], 9[F4M9], and 21[F4M23]. Instructions to follow a specific diet or program of exercise without instructions on how to perform the behaviors are not included in this definition. Cooking and exercise classes, as well as personal trainers and recipes, should always be coded as this technique, but may also be coded as 21[F4M23] (model or demonstrate the behavior)	

[a]BCT: behavior change technique.

[b]CALO-RE: Coventry, Aberdeen, and London-Refined.

[c]SC: smoking cessation.

[d]BCT titles are sourced from Michie et al’s CALO-RE taxonomy [9].

[e]BCT titles are sourced from Michie et al’s smoking cessation taxonomy [10].

References

[9] Michie S, Ashford S, Sniehotta F, Dombrowski S, Bishop A, French D. A refined taxonomy of behaviour change techniques to help people change their physical activity and healthy eating behaviours: the CALO-RE taxonomy. Psychol Health 2011;26(11):1479-98. PMID: 21678185

[10] Michie S, Hyder N, Walia A, West R. Development of a taxonomy of behaviour change techniques used in individual behavioural support for smoking cessation. Addict Behav 2011;36(4):315-9.PMID: 21215528
