# Supplementary material for: Application of Behavior Change Techniques in a Personalized Nutrition Electronic Health Intervention Study: Protocol for the Web-Based Food4Me Randomized Controlled Trial
Source: JMIR Res Protoc. 2018 Apr 9;7(4):e87. doi: 10.2196/resprot.8703 (PMC5913568; doi:10.2196/resprot.8703)
Supplement: Multimedia Appendix 3 [file resprot_v7i4e87_app3.pdf]

Multimedia Appendix 3. Coventry, Aberdeen, and London-Refined (CALO-RE) [9] behavior change techniques (BCTs) excluded from Food4Me, with rationale.

CALO-RE[a] [9] BCT[b] code	BCT description	Reasons for exclusion
12	Prompt rewards contingent on effort or progress toward behavior	Praise, encouragement, and reward were researcher-generated; volunteer participated in intervention
15	Prompting generalization of a target behavior	Beyond scope of study
16	Prompt self-monitoring of behavior	Due to the number of data collection methods used in Food4Me, participants were not asked to keep additional self-monitoring records during the study, only to provide retrospective information at specified time points. As the study was designed to mimic the delivery of a personalized nutrition (PN) service and focused on three kinds of feedback (quantitative self-report, blood indices, and gene-based information), self-monitoring of behavior was not deemed to be a priority in this case.
18	Prompting focus on past success	Beyond scope of study; however, successes were emphasized by researchers in interim reports for participants assigned to PN groups
23	Teach to use prompts or cues	Beyond the scope of study; researcher-generated email reminders were used to prompt participants to take samples and record information; however, no element of teaching or learning was involved with respect to usage of the

		prompt or cue
24	Environmental restructuring	As the study was designed to mimic the delivery of a PN service and focused mainly on the efficacy and frequency four kinds of feedback (quantitative self-report, blood indices, gene-based information, and physical activity [PA]), self-monitoring of behavior was not deemed to be a priority in this case. Beyond scope of study
25	Agree behavioral contract	Advice was provided but participant was not asked to sign up for it specifically
28	Facilitate social comparison	Beyond scope of study
30	Prompt identification as role model or position advocate	Beyond scope of study
31	Prompt anticipated regret	Advice was geared toward emphasizing benefits of improving diet rather than risk
32	Fear arousal	Advice was geared toward emphasizing benefits of improving diet rather than risk, as the consequences of fear arousal would not be possible to control in a remote setting
33	Prompt self-talk	Beyond scope of study
34	Prompt use of imagery	Beyond scope of study
35	Relapse prevention or coping planning	As the study was designed to mimic the delivery of a PN service and focused mainly on the efficacy and frequency of four kinds of feedback (quantitative self-report, blood indices, gene-based information, and PA), relapse prevention or coping planning were not a priority. Beyond scope of study
36	Stress management or emotional	Beyond scope of study

	control training	
37	Motivational interviewing	Beyond scope of study
38	Time management	Beyond scope of study
39	General communication skills training	Beyond scope of study

[a]CALO-RE: Coventry, Aberdeen, and London-Refined.

[b]BCT: behavior change technique.

References

[9] Michie S, Ashford S, Sniehotta F, Dombrowski S, Bishop A, French D. A refined taxonomy of behaviour change techniques to help people change their physical activity and healthy eating behaviours: the CALO-RE taxonomy. Psychol Health 2011;26(11):1479-98. PMID: 21678185

[10] Michie S, Hyder N, Walia A, West R. Development of a taxonomy of behaviour change techniques used in individual behavioural support for smoking cessation. Addict Behav 2011;36(4):315-9.PMID: 21215528
